# Editorial: Emerging talents in pediatric endocrinology: 2022

**DOI:** 10.3389/fendo.2023.1173651

**Published:** 2023-03-08

**Authors:** Willem Staels, Michael A. Levine

**Affiliations:** ^1^ Division of Pediatric Endocrinology, Department of Pediatrics, Universitair Ziekenhuis Brussel (UZ Brussel), Vrije Universiteit Brussel (VUB), Brussels, Belgium; ^2^ Beta Cell Neogenesis (BENE) Research Group, Vrije Universiteit Brussel (VUB), Brussels, Belgium; ^3^ Division of Endocrinology and Diabetes, The Children’s Hospital of Philadelphia, Philadelphia, PA, United States; ^4^ Department of Pediatrics, Perelman School of Medicine, University of Pennsylvania, Philadelphia, PA, United States

**Keywords:** pediatric endocrinology, emerging talent, endocrinology & metabolism, pediatrics, medicine, editorial, special issue, young researchers

The field of pediatric endocrinology has come a long way since its inception by Lawson Wilkins, with basic scientists and medical investigators at the forefront of applying the tools of contemporary biochemistry, cell biology, and genetics to generate new knowledge and translate these discoveries into improved patient care. Further, recent advancements in laboratory techniques and endocrine genetics have expanded the spectrum of diseases managed by pediatric endocrinologists, ranging from disorders that affect growth, pubertal development, sex and gender development, thyroid and adrenal function, to metabolic conditions such as diabetes, obesity, and metabolic bone disease. The primary objective of this special issue, ***“Emerging Talents in Pediatric Endocrinology: 2022”***, is to showcase members of the next generation of investigators in our field and to highlight their early contributions to pediatric endocrinology. These articles describe new research and innovative approaches to disease management that have the potential to improve health outcomes for children and adolescents worldwide. The collection, ***“Emerging Talents in Pediatric Endocrinology: 2022,”*** covers a diverse array of topics in pediatric endocrinology that the reader will find both insightful and significant.

DICER1 syndrome is a pleiotropic tumor-predisposition disorder that significantly increases the risk of thyroid carcinoma, with a standardized incidence ratio of 39, and often presenting before the age of 10 (1). In this Research Topic collection, Ricarte-Filho et al. found that DICER1 mutations in pediatric thyroid cancer disrupt miRNA expression, leading to abnormal cell cycling, reduced thyroid differentiation, and activation of MAPK signaling. These results have implications for potential therapies targeting these pathways.

Patients with congenital hyperinsulinism (CHI) frequently experience recurrent and severe hypoglycemia. Worth et al. developed and tested the application of a Hypoglycemia Error Grid (HEG) to assess the accuracy of Continuous Glucose Monitoring (CGM) in patients with CHI. Their findings suggest that while CGM performance was suboptimal and not recommended as a standalone tool for routine clinical use in CHI, it may have potential use in digital phenotyping of CHI. The HEG is available for other researchers to use in their own assessments of CGM accuracy.

Childhood bone health is important not only for the growing child but also has significant implications for skeletal integrity in adult life. Madhuchani et al. review the use of dual-energy x-ray absorptiometry (DXA) and bisphosphonate therapy in children to rationalize its use in low-middle-income countries. They provide evidence-based guidance for optimally assessing and managing childhood bone disorders in limited resource settings.

Type 1 diabetes (T1D) is a major global health issue with a substantial health and economic burden that is projected to increase rapidly, particularly in resource-limited countries (2). Raicevic et al. conducted a 30-year retrospective study on the incidence of childhood T1D in Montenegro and found a mean annual incidence of 15.2/100,000 person-years. The study suggests that T1D incidence among Montenegrin children is increasing and that the COVID-19 pandemic may have had a short-term influence on new-onset cases.

Obesity is now a global concern that has reached epidemic proportions. Moreover, obesity is the principal risk factor for the development of type 2 diabetes. McGraw et al. show in this issue that the commonly used body mass index (BMI) adjusted for age and sex is an excellent and highly available tool for assessing insulin resistance in Latino children from the Arizona Insulin Resistance registry, compared to newer indices.

As you read this collection, focus not only on the research but also on the researchers. These investigators represent the future of pediatric endocrinology and will drive innovation and improve health outcomes. The implications of this collection for advancing pediatric health outcomes are significant. The breadth and depth of research in pediatric endocrinology demonstrated in this issue highlight its potential to address health disparities, particularly in low-middle-income countries where access to care may be limited.

Contemporary medical research now focuses on the individual as well as the population, with the N-of-1 clinical trial, in which an individual patient is the sole unit of observation, beginning to supplant conventional phase I, II, and III protocols, facilitating accelerated drug evaluation and approval and molecular-based precision medicine. By improving our understanding of the underlying biology of endocrine disorders, we will be able to develop better diagnostic tools and more targeted therapies that will ultimately improve the recognition and treatment of many of the diseases that compromise the optimal growth and development of our patients. Overall, this special issue underscores the importance of research and training in clinical aspects of pediatric endocrinology to ultimately improve health outcomes for all children and adolescents. The contributions of the young researchers in this issue provide a glimpse into the exciting possibilities that the future holds for pediatric endocrinology research and the potential for improving health outcomes for children and adolescents worldwide.

## Author contributions

WS wrote the first draft of the manuscript. MAL revised the manuscript. Both authors read, and approved the submitted version.
